# Supplementary material for: Identification of photoperiod-induced specific miRNAs in the adrenal glands of Sunite sheep (Ovis aries)
Source: Front Vet Sci. 2022 Jul 22;9:888207. doi: 10.3389/fvets.2022.888207 (PMC9354845; doi:10.3389/fvets.2022.888207)
Supplement: Supplementary file 1 [file Data_Sheet_1.ZIP › Supplementary file/miRDeep2 file.zip!/miRDeep2 file/Know_Structure/LP42A1/oar-mir-99a.pdf]

miRBase precursor : oar-mir-99a
Total read count : 718880
oar-miR-99a read count : 718050
remaining reads : 830

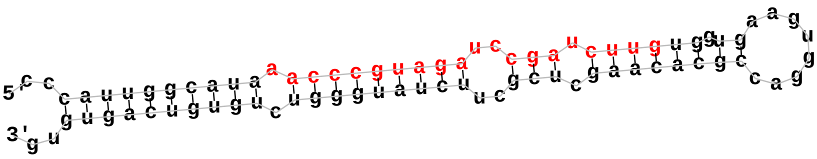

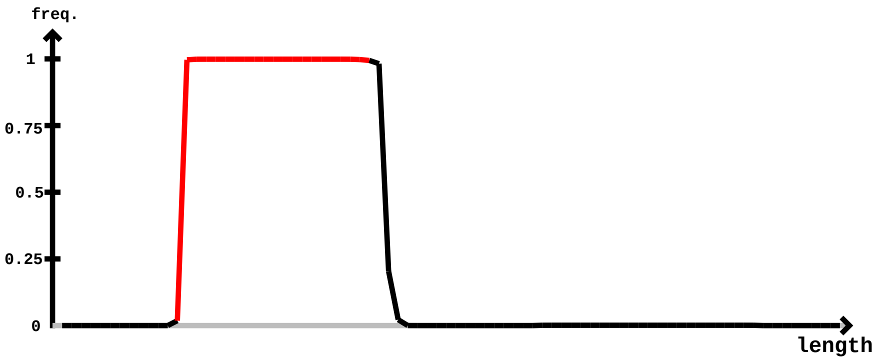

oar-miR-99a

```
5'- cccauuggcauaaacccguagauccgaucuuguggugaaguggaccgcacaagcucgcuucuaugggucugugucagugug   -3'   exp
    ..((((((((((.(((((((((..(((.((((((.((........)))))))).)))..))))))))).))))))))))..   reads      mm      sample
    .........uaaacccguagauccgaucuugu.................................................   1          0       seq
    ..........aaacccguagauccgaucu....................................................   15         0       seq
    ..........aaacccguagauccgaucuu...................................................   62         0       seq
    ..........aaacccguagauccgaucuug..................................................   134        0       seq
    ..........aaacccguagauccgaAcuugu.................................................   5          1       seq
    ..........aaacccguagauccgaucuugu.................................................   12702      0       seq
    ..........aaacccguagauccgaucuugug................................................   305        0       seq
    ..........aaacccguagauccgaAcuugug................................................   2          1       seq
    ..........aaacccguagauccgaucuugugg...............................................   22         0       seq
    ..........aaacccguagauccgaucuuguggu..............................................   1          0       seq
    ...........aacccguagauccgaAc.....................................................   2          1       seq
    ...........aacccguagauccgauc.....................................................   129        0       seq
    ...........aacccgAagauccgauc.....................................................   1          1       seq
    ...........aacccguagauccgaAcu....................................................   76         1       seq
    ...........aacccguagauccgaucu....................................................   769        0       seq
    ...........aacccguagauccgaucuu...................................................   2321       0       seq
    ...........aacccguagauccgaAcuu...................................................   128        1       seq
    ...........aacccguagauccgaucuug..................................................   7497       0       seq
    ...........aacccguagauccgaAcuug..................................................   997        1       seq
    ...........aacccguagauccgaAcuugu.................................................   20292      1       seq
    ...........aacccguagauccgaucuugu.................................................   525462     0       seq
    ...........aacccguagauccgaucuugug................................................   100464     0       seq
    ...........aacccguagauccgaAcuugug................................................   29259      1       seq
    ...........aacccguagauccgaAcuugugg...............................................   525        1       seq
    ...........aacccguagauccgaucuugugg...............................................   15192      0       seq
    ...........aacccguagauccgaAcuuguggu..............................................   6          1       seq
    ...........aacccguagauccgaucuuguggu..............................................   31         0       seq
    ............acccguagauccgauc.....................................................   1          0       seq
    ............acccguagauccgaucu....................................................   2          0       seq
    ............acccguagauccgaAcuu...................................................   1          1       seq
    ............acccguagauccgaAcuug..................................................   13         1       seq
    ............acccguagauccgaucuug..................................................   7          0       seq
    ............acccguagauccgaucuugu.................................................   581        0       seq
    ............acccguagauccgaAcuugu.................................................   196        1       seq
    ............acccguagauccgaAcuugug................................................   567        1       seq
```

oar-miR-99a

```
cccauuggcauaaacccguagauccgaucuuguggugaaguggaccgcacaagcucgcuucuaugggucugugucagugug
.............acccguagauccgaucuugug...............................................    168    0    seq
.............acccguagauccgaucuugugg..............................................    17     0    seq
.............acccguagauccgaAcuugugg..............................................    24     1    seq
.............acccguagauccgaAcuuguggu.............................................    1      1    seq
..............cccguagauccgaucuugu................................................    12     0    seq
..............cccguagauccgaAcuugug...............................................    4      1    seq
..............cccguagauccgaucuugug...............................................    1      0    seq
..............cccguagauccgaucuugugg..............................................    1      0    seq
...............ccguagauccgaucuug.................................................    1      0    seq
...............ccguagauccgaucuugu................................................    30     0    seq
...............ccguagauccgaAcuugug...............................................    1      1    seq
...............ccguagauccgaucuugug...............................................    3      0    seq
................cguagauccgaucuugu................................................    12     0    seq
................cguagauccgaucuugug...............................................    7      0    seq
.................................................caagcucgcuucuaugggucu...........    2      0    seq
.................................................caagcucgcuucuaugggucug..........    20     0    seq
.................................................caagcucgcuucuaugggucugu.........    779    0    seq
.................................................caagcucgcuucuaugggucugug........    3      0    seq
..................................................aagcucgcuucuaugggucugu.........    25     0    seq
.....................................................cucgcuucuaugggucug..........    1      0    seq
```
